# Asn^12^ and Asn^278^: Critical Residues for In Vitro Biological Activity of Reteplase

**DOI:** 10.1155/2010/172484

**Published:** 2010-06-21

**Authors:** Naganath Mandi, Kalyana R. Sundaram, Sunil K. Tandra, Suman Bandyopadhyay, Sriram Padmanabhan

**Affiliations:** ^1^Clone Development Team, Lupin Limited, Biotechnology R & D, Gat #1156, Mulshi Taluka, Ghotawade Village, Pune 411042, India; ^2^Analytical Development Team, Lupin Limited, Biotechnology R & D, Gat #1156, Mulshi Taluka, Ghotawade Village, Pune 411042, India; ^3^Mammalian Bioassay Team, Lupin Limited, Biotechnology R & D, Gat #1156, Mulshi Taluka, Ghotawade Village, Pune 411042, India; ^4^Lupin Limited, Biotechnology R & D, Gat #1156, Mulshi Taluka, Ghotawade Village, Pune 411042, India

## Abstract

Reteplase (rPA) is a thrombolytic agent used for the treatment of acute myocardial infarction. We studied the expression of rPA and its selected asparagine mutants after integration into the *Pichia* genome. Though methanol induction of the native and the rPA mutants showed similar expression levels (~200–250 mg/L), the mutants displayed significant loss of protease activity. Strikingly, the clot lysis activities of these mutants were considerably different. While mutation of Asn^12^ (N12P) of the Kringle 2 domain showed delayed clot lysis activity (*t*_1/2_ = 38 min) compared to the native rPA (*t*_1/2_ = 33 min), a faster rate of clot lysis (*t*_1/2_ = 27 min) was observed when the Asn^278^ (N278S) of the serine protease domain was mutated. Interestingly, the slowest clot lysis activity (*t*_1/2_ = 49 min) demonstrated by the double mutant (N12P, N278S) suggests the dominant role of Asn^12^ in regulating the fibrinolytic activity of rPA. The results presented in this paper indicate that the fibrinolytic and the proteolytic activities of rPA are independent of each other.

## 1. Introduction

Thrombolysis is the first choice of therapy for acute myocardial infarction (AMI). The most common thrombolytic agents (TAs) include streptokinase (first generation TA) and alteplase (second generation TA, tissue plasminogen activator, tPA). The third generation TAs' like rPA, tenecteplase and lanoteplase, are some of the derivatives of alteplase. Human tPA is a 70 kDa serine protease with two forms Type I tPA and Type II tPA with major differences in their core glycosylation. There are four potential *N*-linked glycosylation sites for tPA at positions Asp-117, -184, -218, and -448. 

The tPA molecule contains five discrete structural domains. In the presence of plasmin, single-chain tPA, or zymogen enzyme can be cleaved into an activated two-chain form. The heavy chain contains four of these domains: a “finger” domain which is homologous to a portion of fibronectin; a “growth factor” domain which is homologous to epidermal growth factor; two nonequivalent “Kringle” domains. The light chain contains the serine protease domain, which is homologous to trypsin and chymotrypsin. It is reported that plasmin cleavage occurs C-terminal to Kringle 2 (at Arg278) to form two-chain tPA [1]. 

Reteplase (recombinant plasminogen activator, rPA) is a thrombolytic agent approved by the Food and Drug Administration (http://en.wikipedia.org/wiki/Food_and_Drug_ Administration) in 1996 for the management of acute myocardial infarction (AMI) in adults, in the reduction of the incidence of congestive heart failure, and for reduction of mortality associated with AMI. It is a single-chain deletion mutant of tPA containing C-terminal kringle 2 and serine protease domains of tPA which includes, 355 amino acids with a total molecular weight of 39 kDa. The protease domain is responsible for converting plasminogen to plasmin, which in turn degrades the fibrin matrix of thrombus while the kringle 2 domain binds specifically to the fibrin clot. Because of the deletion of the fibronectin finger region, the binding of rPA to fibrin is significantly reduced in comparison with that of alteplase. In clinical trials, rPA has demonstrated more rapid and complete coronary patency compared with alteplase, without significant increase in the clinical adverse events [2, 3]. 

The rPA molecule is a nonglycosylated protein form of human tPA efficiently produced in *E. coli *containing 1–3 and 176–527 amino acids of tPA. Such an engineered rPA molecule carries three potential *N*-glycosylation sites at Asn-184, -218 and -448 with a consensus sequence Asn-Xaa-Ser/Thr from human tPA. The asparagine positions in the rPA are Asn-12, -48, and -278, respectively. 

rPA has been expressed in various expression hosts like* Bacillus subtilis* [4], seaweed of *Laminaria japonica* [5], mammalian system [6–8], yeast like *Pichia methanolica* [9], *Pichia pastoris* [10, 11], fungal systems [12], and insect cells [13], and so forth. The expression levels of rPA between all these systems vary considerably with problems of protein instability. While a lot of literature exists on expression of rPA in bacterial system like *E. coli* [14–16], the method involves long and inefficient renaturation procedures [17] with poor recovery during downstream operations [18].

*Pichia pastoris* is a well-known yeast used by researchers worldwide for expression of proteins in soluble form [19–22] due to its advantages like robustness, growth in chemically defined media, shorter fermentation times as compared to mammalian system, effective glycosylation, absence of endotoxins, achieving high-cell density, and so forth. Although reports on expression of rPA in methylotrophic yeast *Pichia pastoris *are available, the yield is merely 100 mg/L by conducting fermentation studies at the usual temperature of 30°C [11]. A further lower temperature of 20°C during fermentation was found to enhance the expression levels to 200 mg/L [11]. Addition of glycerol to the cultures has been reported to enhance the levels of rPA expression to nearly 200 mg/L when the fermentation is carried out at 30°C [23]. Zou and Chu [24] have studied the host's enzyme activity during rPA production phase by recombinant *Pichia pastoris* with not much stress on expression levels. 

In the present paper, we report the successful generation of *Pichia* recombinants carrying multiple copies of *rPA* gene integrated into *Pichia* genome by homologous recombination. Such high-geneticin-resistant *Pichia pastoris *recombinants expressed rPA to reasonably appreciable level of 250 mg/L as judged by ELISA in shake flask studies. Moreover, one could observe stable expression of rPA during the entire induction period of 72 h. Considering the significance of asparagine residues of *N*-linked glycosylation sites in maintaining structural integrity and catalytic function of proteases [25, 26], we attempted to investigate the role of asparagines involved in the *N*-linked glysosylation sites present in activity regulating domains of rPA. When the native *rPA* gene was subjected to site directed mutagenesis (SDM) to modify the potential asparagine linked glycosylation sites, we found dramatic reduction in the protease activity of all the mutants with insignificant changes in their expression levels. Interestingly, the rate of clot lysis was enhanced by 20% when the mutation was in the protease domain while it reduced by 20% when the asparagine mutation was in the kringle 2 domain of rPA.

## 2. Materials and Methods

### 2.1. Yeast Strains, Plasmids, Enzymes and Reagents

*Escherichia coli* DH5*α*, PCR reagents, restriction and ligation enzymes, SDS-PAGE reagents were procured from Bangalore Genei, Bangalore (India). Oligos, gel purification kit and PCR purification kits were obtained from Sigma (USA). Plasmid pPIC9K, *Pichia pastoris* strain GS115, *Pichia *expression kit (Spheroplast module) and geneticin were purchased from Invitrogen (Carlsbad, CA USA). Luria Bertani broth (LB), LB agar, yeast extract, bacto peptone, yeast nitrogen base (YNB) without amino acids were from BD (USA). Dextrose, sorbitol, amino acids and D-biotin for selection of *Pichia* transformants were procured from Sigma (USA). Site directed mutagenesis kit (Quick Change II ) was obtained from Stratagene, USA. Chromozym t-PA substrate (N-Methylsulfonyl-D-Phe-Gly-Pro-Arg-4 nitroanilide acetate) was procured from Roche, Germany while human plasminogen, human fibrinogen and human thrombin were from Sigma, USA. The chromogenic substrate D-Val-Leu-Lys 4-nitroanilide dihydrochloride used for plasminogen activation assay was purchased from Sigma, USA. The polyclonal rabbit anti-rPA IgG and goat antirabbit IgG-HRP antibodies were purchased from Abexome Biosciences, India.

### 2.2. PCR Amplification and Cloning of rPA Gene

*rPA* gene was custom synthesized at GenArt (Germany) and used as a template for PCR amplification. The PCR was carried out in 50 *μ*L volume with forward primer 5′ GGG GTA TCT CTC GAG AAA AGA TCT TAC CAA GGC AAC AGC GAT 3′ and reverse primer 5′ CCG CCG GAA TTC TAC GTA TTA GGC TCG CAT GTT GTC ACG AAT 3′ using two step PCR programme: initial denaturation at 94°C for 4 min followed by 5 cycles of 94°C for 30 sec, 50°C for 30 sec, and 72°C for 90 sec; and 25 cycles of 94°C for 30 sec, 60°C for 30 sec and 72°C for 1 min with final extension 7 min at 72°C. The 1.1 kb PCR product was digested with *Xho*I and *Sna*BI enzymes and cloned into pPIC9K cut with *Xho*I/*Sna*BI enzymes. The clone development was carried out in DH5*α* cells. The clones were confirmed by restriction analysis and DNA sequencing.

### 2.3. SDM of rPA Gene at Asparagine Residues

The oligos designed for the mutation of asparagine residues are shown in Table 1. Briefly, the pPIC9K-rPA DNA was subjected to site directed mutagenesis using Stratagene's Quick Change kit. The PCR was carried out in 50 *μ*L volume and the PCR conditions were according to manufacturer's protocol. After the PCR, the mix was treated with *Dpn*I for 1 h at 37°C and used to transform the DH5*α* cells. The mutant clones were selected by restriction analysis. Asn^12^ and Asn^278^ residues which are potential sites of *N*-linked glycosylation were selected for SDM.

### 2.4. SDM Confirmation by Restriction Analysis

The clone carrying mutation at Asn^12^ of the *rPA* gene created Sma1 site (N12P), the Asn^278^ mutation created a *Xba*I site (N278S). The clone carrying double mutation at Asn^12^ and Asn^278^ contained both *Xba*I and *Sma*I site (N12P, N278S). The clones carrying mutations of rPA gene were subjected to PCR separately using AOX primers as described above and the purified PCR products were subjected to restriction digestion using appropriate restriction endonucleases for easy identification of successful SDM.

### 2.5. Transformation of P. pastoris Strain GS115 with Linearized DNA of pPIC9K-rPA and Its Mutants and Selection of Geneticin Resistant Clones

Spheroplasting of *P. pastoris* strain GS115 was carried out using *Pichia* expression kit by following manufacturer's protocol (Invitrogen, CA, USA). Spheroplasted GS115 cells were transformed with 10 *μ*g of the *SalI *linearized pPIC9K-rPA plasmid and the transformants were plated on histidine deficient RDB (containing 1.34% (w/v) yeast nitrogen base, 2% (w/v) dextrose, 1 M Sorbitol, 0.005% (w/v) amino acids, 4 × 10^−5^% (w/v) biotin and 2% (w/v) agar) medium for the selection for His^+^ phenotypes. All His^+^ transformants were picked up and grown in YPD broth for 48 hrs at 30°C and were then spotted on YPD agar plates with geneticin at three different concentrations, namely, 0.5 mg/mL, 1 mg/mL, and 2 mg/mL. There are extensive reports of hyper expression of foreign proteins from higher geneticin resistant *Pichia* transformants, since such transformants carry high copy numbers of the integrated genes [7, 8]. Transformants with highest geneticin resistance, which carry multiple copies of the integrated rPA gene, were further confirmed for rPA gene insertion by colony PCR using *AOX1* sequencing primers (Invitrogen, CA, USA). A typical PCR reaction was set up in a 25 *μ*L reaction volume that consisted of initial denaturation at 95°C for 5 min followed by 30 cycles of 95°C for 30 sec, 55°C for 30 sec, and 72°C for 1 min with a final extension at 72°C for 7 min. Clones showing right size PCR product and highest geneticin resistance were chosen for expression studies.

### 2.6. Expression of Native rPA and Its Mutants in Shake Flask

Single colony from each of the transformants was inoculated into 50 mL BMGY (buffered complex glycerol medium) separately and grown at 30°C for 48 h. The cultures were centrifuged at 4000 rpm for 10 min and pellets were resuspended in 10 mL BMMY (buffered complex methanol medium). The methanol was added at 1% final concentration daily for three days. The expression of rPA was checked by running supernatant fraction on 12% SDS-PAGE and visualized by silver staining.

### 2.7. Quantitation of rPA in Pichia Supernatant by ELISA

The supernatant from the induced *Pichia* culture was dialyzed against 10 mM Tris pH 7.6 and used for the quantitation of rPA by direct ELISA. The primary and secondary antibodies were rabbit anti-rPA IgG and goat antirabbit IgG-HRP, respectively, and the substrate was TMB. The yield of the rPA was calculated using retavase as standard and expressed as mg/L.

### 2.8. Biological Activity of the rPA

#### 2.8.1. Protease Assay

Enzymatic activity of rPA was monitored by direct cleavage of the chromogenic substrate Chromozym t-PA (N-Methylsulfonyl-D-Phe-Gly-Pro-Arg-4 nitroanilide acetate) as per manufacturer's protocol with some modifications as described below. Briefly, 6.25 *μ*L of samples were incubated with 62.5 *μ*L of 9-fold diluted chromozym t-PA solution (4 mM) in 96-well plate and incubated for 30 minutes at room temperature in dark. 100 mM Tris, pH 8.5 served as reagent blank. The substrate was cleaved by rPA resulting in the formation of yellow colored 4-nitraniline (pNA) which was measured at 405 nm after stopping the reaction using 31.25 *μ*L citric acid (10%, w/v). Blank subtracted absorbance was represented as relative activity of the samples [27]. Bacterial derived rPA (retavase) was used as a positive control and for comparison studies.

#### 2.8.2. Clot Lysis Assay

Fibrin gel (100 *μ*L/reaction) was prepared in a 96-well flat bottom plate by incubating a mixture of human fibrinogen (250 *μ*g), human plasminogen (1 *μ*M), and human thrombin (1 U) per reaction at 37°C for 30 min. The dissolution of the clots was monitored at 340 nm by a plate reader (Multiskan Spectrum, Thermo) at different time points (0, 15, 30, 45, 60 min) after addition of 100 *μ*L of *Pichia* culture supernatants on the surface of the clot. The plate was incubated at 37°C for the specified time period and continuously shaken at 400 rpm. The % fibrin gel lysis was calculated by extrapolating the readings of the respective wells at O.D. 340 nm considering the reading of the well containing medium only and without thrombin as 100% clot lysis. The fibrinolytic activity of the samples was quantitated by the lysis time (*t*_1/2_) which is the time needed to reduce the turbidity of the clot to the half-maximal value [28].

#### 2.8.3. Plasminogen Activation Assay

Plasminogen activation by rPA was determined by a chromogenic assay as previously described [19]. Briefly, 25 mU of human plasminogen, chromogenic substrate D-Val-Leu-Lys 4-nitroanilide dihydrochloride and samples containing rPA were incubated in 96-well flat-bottom plates (Nunc) at 25°C for 30 min. rPA activates plasminogen to form plasmin, which in turn cleaves the chromogenic substrate to release yellow coloured pNA which was measured at 405 nm by a plate reader.

## 3. Results

### 3.1. Construction of pPIC9K-rPA Clone

**Figure 1:**
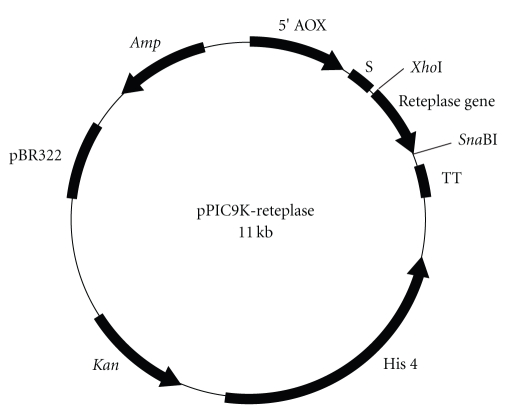
Plasmid map of pPIC9K-rPA.

The *rPA* gene sequence includes 1–3 amino acids of the finger domain and 176–527 amino acids of K2S domains of the tPA molecule. The clone was confirmed by restriction analysis and DNA sequencing. The plasmid map constructed is shown in Figure 1.

### 3.2. Confirmation of SDM for rPA Mutants

**Figure 2:**
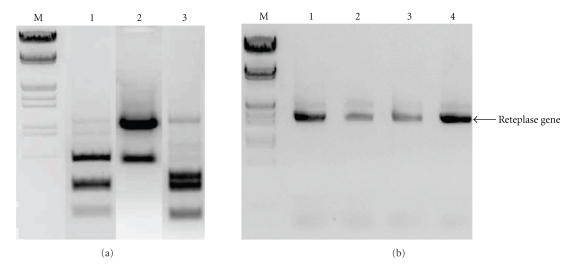
(a) Restriction analysis of the PCR product of pPIC9K-rPA mutated constructs. M: lambda *EcoR1*/*HindIII* digest, lane 1: PCR product from N12P clone+ *Sma1*; lane 2: N278S clone+ *XbaI*; lane 3: N12P, N278S +*Xba1/Sma1*. (b) Integration PCR of *Pichia *recombinants carrying native rPA gene. M: lambda *EcoR1*/*HindIII* digest; lanes 1–4 geneticin resistant clones of *Pichia* carrying ppIC9K-reteplase gene and its mutants integrated into its genome. Lane 1: N12P clone; lane 2: N278S; lane 3; N12P, N278S; lane 4: WT reteplase.

The PCR product of reteplase gene isolated from pPIC9K-rPA construct and its SDM mutants were examined for the digestion with the respective restriction endonucleases. The results (Figure 2(a)) indicate that *Sma*I digestion of N12P gave DNA fragments of expected sizes, namely, 750 bp, 400 bp, 386 bp, and 260 bp while N278S clone yielded two fragments of 1184 bp and 440 bp upon *Xba*I digestion.

### 3.3. Transformation of GS115 Cells and Expression of Native rPA Molecule

*Pichia pastoris* host GS115 cells were transformed with linearized pPIC9K-rPA DNA by spheroplasting method. The *Pichia* recombinant which showed resistance to 2 mg/mL geneticin was selected for rPA expression in shake flasks. Initial expression of rPA in the BMMY medium with 1% methanol was very less (data not shown). However, this expression was improved to higher yield by addition of Tween-80 to a final concentration of 0.1%. The molecular weight of recombinant native rPA (WT) was ~55 kDa. This was further confirmed by Western blot studies (data not shown).

### 3.4. Generation of Pichia Recombinants Carrying Asaparagine Substitutions and Analysis

Asn^12^ and Asn^278^ residues which are the two potential sites of *N*-linked glycosylation were selected for SDM. These asparagine residues were changed to proline and serine, respectively, just to enable creation of a restriction site for further confirming the mutation reaction. The His^+^ geneticin resistant colonies were subjected to integration PCR using *AOXI* specific primers as per the protocol described by Apte-Deshpnade et al. [19]. The results in Figure 2(b) show successful integration of *rPA* gene into *Pichia* genome as evident by the right size PCR product.

### 3.5. Amino Acid Sequence Comparisons between WT rPA and Its Mutants

**Figure 3:**
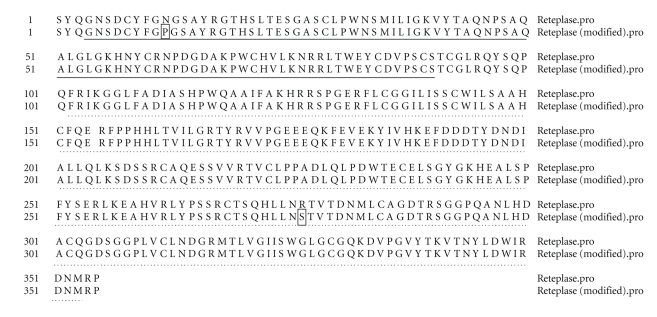
Amino acid sequence comparisons between native and rPA SDM mutants. Bold line indicates kringle 2 domain while dotted line indicates the serine protease domain. The asaparagine substitutions have been boxed. Reteplase refers to WT rPA gene while modified reteplase refers to mutated rPA gene.

The amino acid sequence comparisons between native and asparagine rPA mutants is depicted in Figure 3. The data shows the modified asparagine residues in the mutants studied.

### 3.6. Expression of Recombinant rPA from P. pastoris in Shake Flasks

**Figure 4:**
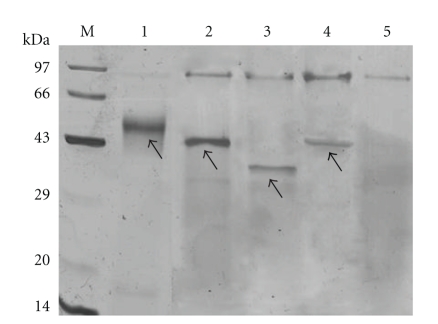
SDS-PAGE analysis of *Pichia *derived native rPA and its mutants. M: Medium protein molecular weight marker (97 to 14 kDa); lane 1: native rPA (~55 kDa); lane 2: (N12P mutant ~43 kDa); lane 4: (N12P, N278S mutant, ~39 kDa); lane 4: (N278S, ~43 kDa) lane 5: GS115 control.

The predicted molecular weight of the native rPA is 39 kDa while on SDS-PAGE it showed a diffused band with a molecular weight of ~55 kDa (Figure 4). This is expected since glycosylated proteins are known to exhibit higher molecular weight on SDS-PAGE. The SDS-PAGE for supernatants from all the SDM mutants also showed similar expression levels. The Asp-48 was not chosen for modification for this study since the consensus sequence of the tripeptide acceptor sequeon is Asn-Pro-Thr, where proline is known to prevent the *N*-linked glycosylation [1]. The other mutations did show reduced *N*-linked glycosylation since the proteins showed a clear band (Figure 4) of expected sizes, 43 kDa for N12P and N278S and 39 kDa for the double mutant (N12P, N278S).

### 3.7. Quantitation of rPA by ELISA

**Figure 5:**
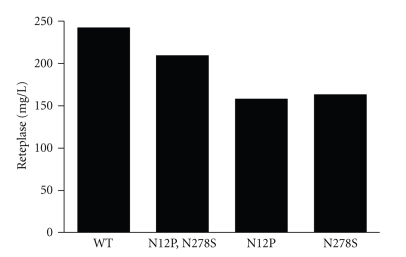
ELISA for rPA quantitation in *Pichia* fermentation supernatants. *X* axis shows sample types, *Y* axis is rPA concentration (mg/L).

The expression levels of the rPA from the native as well as the SDM mutants were estimated by ELISA using anti-rPA polyclonal antibody. The amount of rPA expressed correlated well with the observations of SDS-PAGE and the yields of the native and its mutants showed expression levels in the range of 210–250 mg/L (Figure 5).

### 3.8. Biological Activity of rPA and Its Mutants

#### 3.8.1. Protease Activity by Chromozym t-PA Assay

**Figure 6:**
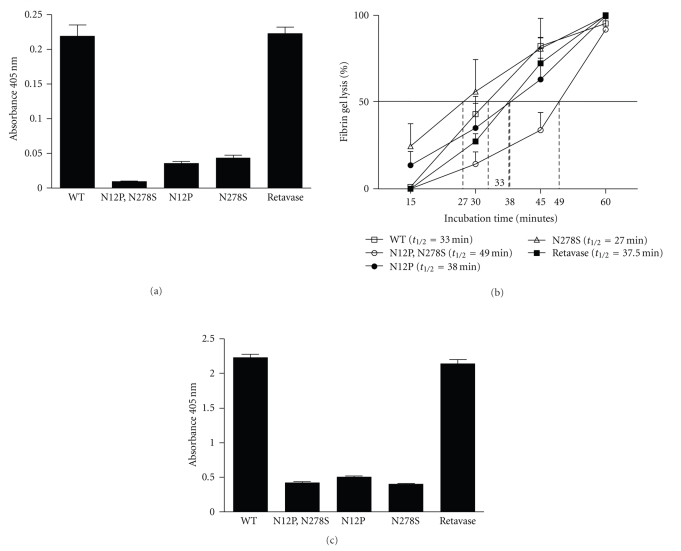
(a) Protease activity assay for rPA and its mutants. Sample 1 is WT (wild type rPA gene); sample 2: N12P, N278S double mutant; sample 3: N12P mutant; sample 4: N278S mutant; sample 5: commercial retavase preparation. (b) Clot lysis activity by turbidimetric fibrinolytic assay. *X* axis shows incubation time in minutes for clot lysis while *Y* axis shows % clot lysis. The horizontal line indicates 50% clot lysis while the vertical dotted lines indicate the *t *_1/2_ values of different rPA samples. The course of clot lysis is demonstrated by connecting data points of different time intervals. (c) Plasminogen activation assay. *X* axis shows sample types while *Y* axis shows absorbance at 405 nm. Sample 1: WT rPA; sample 2: N12P, N278S double mutant; sample 3: N12P mutant; sample 4: N278S mutant; sample 5: commercial retavase preparation.

The native rPA clone (WT) showed protease activity as commercially available rPA preparation (retavase), the clones carrying asparagine mutations (N12P, N278S and N12P, N278S) lost their protease activity significantly (*P* < .0001). Notably, the activity was significantly decreased (*P* < .001) in the double-mutated rPA molecule (N12P, N278S) compared to that of single mutated clones like N12P or N278S (Figure 6(a)).

#### 3.8.2. Clot Lysis Activity Assay by Turbidimetric Fibrinolytic Assay

Activity of rPA was represented as the rate of clot lysis by turbidimetric fibrinolytic assay. Similar to Chromozym t-PA assay, double mutated rPA clone (N12P, N278S) resulted in most delayed activity (*t*_1/2_ = 49 mins) as compared to the WT rPA of (*t*_1/2_ = 33 min). Very interestingly, single mutation of the Asn^12^ (N12P) of the Kringle 2 domain, known for its fibrin selectivity, also showed delayed activity (*t*_1/2_ = 38 min), presumably indicating the loss of fibrin-binding. However, the fibrinolytic activity of N278S (*t*_1/2_ = 27 min) remain unaffected indicating the noncritical nature of this asparagine for clot lysis. It is clear from the Figure 6(b) that *Pichia*-derived crude rPA variant (N278S) is better than retavase as far as clot lysis activity is concerned supporting our earlier observations of chromozym protease assay. This data clearly indicate that fibrinolytic activity and proteolytic activity of rPA is independent of each other.

#### 3.8.3. Plasminogen Activation Assay

Similar to our previous observations, the mutation of Asn^12^ and Asn^278^ significantly reduced the plasminogen activation property of rPA by nearly 3- to 4-fold, (*P* = .0002) as depicted in Figure 6(c). The plasminogen binding activity of retavase was found to be similar to crude *Pichia* rPA (Figure 6(c)). This result indicates that the plasminogen binding property of the serine protease domain of rPA is indeed affected by asparagine mutation.

## 4. Discussion

Yeast is often preferred for expression of recombinant proteins from higher eukaryotes. Like bacteria, yeast cultivation is fast at low cost and a large variety of techniques exist for the production and manipulation of the proteins produced. Yeast, however, possess the specific advantage over bacterial production systems in that they provide an environment for posttranslational modification and secretion (e.g. acylation, phosphorylation, glycosylation, formation of disulfide bonds). These modifications are often essential for the function and/or stability of the expressed protein. Although yeast-like *Kluyveromyces lactis* has been employed of rPA expression, the yield is rather low as suggested [17]. Since it is claimed that the pharmacokinetic properties of a tPA variant is better with deglycosylated version [16], it seems imperative that our present study assumes critical importance. 

Methylotrophic yeast has gained increasing interest for fundamental research and as attractive hosts for the production of biologically active proteins [29, 30]. Although, the nonglycosylated, truncated tPA (rPA, Retavase) produced in *E. coli *in form of inclusion bodies is biologically active form available commercially for therapeutic use, our present data on loss of protease activity and enhanced clot lysis activity obtained with Asp-278 mutation highlights the criticality of this asparagine residue of rPA for the first time with no effect on its expression levels. On the other side, the asparagine mutation of the kringle 2 domain reduced both the protease and the clot lysis activity significantly and this observation is being reported for the first time. 

The observations of complete loss of protease activity seen with the rPA double mutant along with the slowest clot lysis activity (*t*_1/2_ = 49 min) as compared to the single mutated rPA clones like N12P or N278S reiterates the dominant role played by the asparagine residue of the kringle 2 domain in regulating the fibrinolytic activity of rPA.

Work by Collen et al. [31] have shown the increased catalytic efficiency for plasminogen activation by site directed mutagenesis of tPA in the kringle domains and recently a paper by Lee et al. [32] showed antiendothelial property of just the kringle 1 and 2 domains in *Pichia pastoris* with no glycosylation are research work carried out on similar lines. This fibrin affinity is believed to be due to interactions of the finger and kringle 2 domain with fibrin. For reasons poorly understood, kringle 1 domain interacts more weakly than kringle 2 with fibrin [33]. The different affinities might be due to different orientations of the domains relative to the serine protease domain. Certain amino acid residues in the plasminogen-binding region of the tPA protease domain provide favorable binding energies for portions of the plasminogen molecule and that these interactions can be increased by substitution of different amino acids at these positions [33].

The reduced plasminogen activity for all the rPA mutants also supports the claims of the residence of the plasminogen binding domain in the protease domain of tPA [34]. Our observations of differential effect of asparagine mutations on protease and clot lysis activity correlates with similar observations of Wang et al. [35] for another plasminogen activator molecule urokinase. It is possible to speculate that the Asn^278^ substitution with serine has modified the topology of the rPA molecule for enhanced fibrin binding and thereby enhanced clot lysis. Our data supports the observations of Paoni et al. [36] that substitutions of the protease domain of tissue plasminogen activator at four sites including I^275^ enhanced fibrin binding.

Our results on loss of serine protease activity of rPA upon asparagines substitutions support the observations of a recent report on role of asparagine-linked glycosylation site on matriptase, a transmembrane serine protease [26] and contribution of asparagines in maintaining the structural integrity of a cysteine protease-like papain [25].

Our present data reflecting high yields of rPA to a tune of 250 mg/L in shake flask is comparable to literature reports, although in a nonoptimized medium. This could be attributed to high copy number of the *rPA* gene integrated into the *Pichia* genome. It is worth mentioning at this stage that the rPA expression was seen on all the fermentation days without any signs of degradation and this is in contrast to the previous observations of Shu-guang et al. [11] who reported degradation of *Pichia* derived rPA during routine fermentation conditions.

It is worth mentioning here that the activity observed for the crude *Pichia* derived native rPA is comparable to bacterial-derived commercial rPA preparation and thus hints towards the potential possibility of achieving better rPA preparations from *Pichia*. Moreover, achieving better clot lysis activity with the present rPA mutants opens up the possibility of development of next generation reteplase molecules for improved therapeutic use.

## Figures and Tables

**Table 1:** Oligos used for carrying out SDM of wild type (native) rPA gene.

Asn^12^	FP: 5′ GGC AAC AGC GAT TGC TAT TTT GGC CCC GGG AGC GCG TAT CGC GGC ACC 3′
	RP: 5′ GGT GCC GCG ATA CGC GCT CCC GGG GCC AAA ATA GCA ATC GCT GTT GCC 3′

Asn^278^	FP: 5′ GC ACA TCA CAA CAT TTA CTT TCT AGA ACA GTC ACC GAC AAC 3′
	RP: 5′ GTT GTC GGT GAC TGT TCT AGA AAG TAA ATG TTG TGA TGT GC 3′

## References

[1] Pennica D, Holmes WE, Kohr WJ (1983). Cloning and expression of human tissue-type plasminogen activator cDNA in *E. coli*. *Nature*.

[2] Nordt TK, Bode C (2003). Thrombolysis: newer thrombolytic agents and their role in clinical medicine. *Heart*.

[3] Ellis K, Brener S (2004). New fibrinolytic agents for MI: as effective as current agents, but easier to administer. *Cleveland Clinic Journal of Medicine*.

[4] Wang L-F, Hum WT, Kalyan NK, Lee SG, Hung PP, Doi RH (1989). Synthesis and refolding of human tissue-type plasminogen activator in *Bacillus subtilis*. *Gene*.

[5] Zhang Y, Jiang P, Gao J (2008). Recombinant expression of rt-PA gene (encoding Reteplase) in gametophytes of the seaweed *Laminaria japonica*. *Science in China, Series C*.

[6] Spellman MW, Basa LJ, Leonard CK (1989). Carbohydrate structures of human tissue plasminogen activator expressed in Chinese hamster ovary cells. *Journal of Biological Chemistry*.

[7] Parekh RB, Dwek RA, Rudd PM (1989). N-glycosylation and in vitro enzymatic activity of human recombinant tissue plasminogen activator expressed in Chinese hamster ovary cells and a murine cell line. *Biochemistry*.

[8] Li X-K, Lijnen HR, Nelles L, Van Hoef B, Stassen JM, Collen D (1992). Biochemical and biologic properties of rt-PA del(K296-G302), a recombinant human tissue-type plasminogen activator deletion mutant resistant to plasminogen activator inhibitor-1. *Blood*.

[9] Jie J, Cheng-Ying J, Lian-Xiang D (2006). Cloning of rPA gene and expression of the gene in *Pichia methanolica*. *Journal of South China University and Technolology*.

[10] Shu-guang F, Ju C, Li H, Ying-ping Z, Si-liang Z (2006). Degradation of reteplase expressed by recombinant *Pichia pastoris*. *Journal of East China University of Science and Technology*.

[11] Shu-guang F, Huang CJ, Ying-ping Z, Si-liang (2007). Effects of temperature on expression of rPA in *Pichia pastoris*. *Industrial Microbiology*.

[12] Wiebe MG, Karandikar A, Robson GD (2001). Production of tissue plasminogen activator (t-PA) in *Aspergillus niger*. *Biotechnology and Bioengineering*.

[13] Farrell PJ, Behie LA, Iatrou K (1999). Transformed lepidopteran insect cells: new sources of recombinant human tissue plasminogen activator. *Biotechnology and Bioengineering*.

[14] Dormiani K, Khazaie Y, Sadeghi HMM, Rabbani M, Moazen F (2007). Cloning and expression of a human tissue plasminogen activator variant: K2S in *Escherichia coli*. *Pakistan Journal of Biological Sciences*.

[15] Mattes R (2001). The production of improved tissue-type plasminogen activator in *Escherichia coli*. *Seminars in Thrombosis and Hemostasis*.

[16] Manosroi J, Tayapiwatana C, Götz F, Werner RG, Manosroi A (2001). Secretion of active recombinant human tissue plasminogen activator derivatives in *Escherichia coli*. *Applied and Environmental Microbiology*.

[17] Martegani E, Forlani N, Mauri I, Porro D, Schleuning WD, Alberghina L (1999). Expression of high levels of human tissue plasminogen activator in yeast under the control of an inducible GAL promoter. *Applied Microbiology and Biotechnology*.

[18] Zhao Y, Ge W, Kong Y, Zhang C (2003). Cloning, expression and renaturation studies of reteplase. *Journal of Microbiology and Biotechnology*.

[19] Apte-Deshpnade A, Mandal G, Soorapaneni S, Prasad B, Kumar J, Padmanabhan S (2009). High-level expression of non-glycosylated and active staphylokinase from *Pichia pastoris*. *Biotechnology Letters*.

[20] Roy N, Padmanabhan S, Smith M, Shi L, Navre M, Das G (1999). Expression of human gelatinase B in *Pichia pastoris*. *Protein Expression and Purification*.

[21] Cereghino JL, Cregg JM (2000). Heterologous protein expression in the methylotrophic yeast *Pichia pastoris*. *FEMS Microbiology Reviews*.

[22] Cregg JM, Vedvick TS, Raschke WC (1993). Recent advances in the expression of foreign genes in *Pichia pastoris*. *Nature Biotechnology*.

[23] Shu-guang F, Chu J, Huang CJ, Ying-ping Z, Si-liang Z (2007). Effects of glycerol feeding strategies on production of rPA from *Pichia pastoris*. *Industrial Microbiology*.

[24] Zou M, Chu J (2008). Enzyme activity during reteplase production phase by recombinant *Pichia pastoris*. *Journal of Southern Medical University*.

[25] Vernet T, Tessier DC, Chatellier J (1995). Structural and functional roles of asparagine 175 in the cysteine protease papain. *Journal of Biological Chemistry*.

[26] Miyake Y, Tsuzuki S, Mochida S, Fushiki T, Inouye K (2010). The role of asparagine-linked glycosylation site on the catalytic domain of matriptase in its zymogen activation. *Biochimica et Biophysica Acta*.

[27] Verheijen JH, de Jong YF, Chang GTG (1985). Quantitative analysis of the composition of mixtures of one-chain and two-chain tissue-type plasminogen activator with a spectrophotometric method. *Thrombosis Research*.

[28] Jin T, Bokarewa M, Zhu Y, Tarkowski A (2008). Staphylokinase reduces plasmin formation by endogenous plasminogen activators. *European Journal of Haematology*.

[29] Gellissen G, Weydemann U, Strasser AWM, Piontek M, Janowicz ZA, Hollenberg CP (1992). Progress in developing methylotrophic yeasts as expression systems. *Trends in Biotechnology*.

[30] Hollenberg CP, Gellissen G (1997). Production of recombinant proteins by methylotrophic yeasts. *Current Opinion in Biotechnology*.

[31] Collen D, Lijnen HR, Bulens F, Vandamme A-M, Tulinsky A, Nelles L (1990). Biochemical and functional characterization of human tissue-type plasminogen activator variants with mutagenized kringle domains. *Journal of Biological Chemistry*.

[32] Lee S-B, Oh H-K, Kim H-K, Joe YA (2006). Expression of the non-glycosylated kringle domain of tissue type plasminogen activator in *Pichia* and its anti-endothelial cell activity. *Protein Expression and Purification*.

[33] van Zonneveld AJ, Veerman H, MacDonald ME, van Mourik JA, Pannekoek H (1986). Structure and function of human tissue-type plasminogen activator (t-PA). *Journal of Cellular Biochemistry*.

[34] Livingston DJ, Parthasarathy M Gene sequences encoding modified residues situated in the protease domain of tPA.

[35] Wang P, Zhang J, Sun Z, Chen Y, Gurewich V, Liu J-N (2000). Catalytic and fibrinolytic properties of recombinant urokinase plasminogen activator from *E. coli*, mammalian, and yeast cells. *Thrombosis Research*.

[36] Paoni NF, Chow AM, Peña LC, Keyt BA, Zoller MJ, Bennett WF (1993). Making tissue-type plasminogen activator more fibrin specific. *Protein Engineering*.

